# Patient demographics and characteristics from an ambispective, observational study of patients with duchenne muscular dystrophy in Saudi Arabia

**DOI:** 10.3389/fped.2022.1020059

**Published:** 2022-09-30

**Authors:** Abdulaziz S. AlSaman, Fouad Al Ghamdi, Ahmed K. Bamaga, Nahla AlShaikh, Mohammed Al Muqbil, Osama Muthaffar, Fahad A. Bashiri, Baleegh Ali, Arzu Mulayim, Elena Heider, Abdullah A. Alshahrani, Mohammed A. Al Muhaizea

**Affiliations:** ^1^Pediatric Neurology Department, National Neuroscience Institute, King Fahad Medical City, Riyadh, Saudi Arabia; ^2^Neuroscience Center, King Fahad Specialist Hospital, Dammam, Saudi Arabia; ^3^Neurology Division, Department of Pediatrics, Faculty of Medicine, King Abdulaziz University Hospital, King Abdulaziz University, Jeddah, Saudi Arabia; ^4^Department of Pediatrics, King Faisal Specialist Hospital and Research Centre, Jeddah, Saudi Arabia; ^5^Department of Pediatrics, Ministry of National Guard Health Affairs, Jeddah, Saudi Arabia; ^6^College of Medicine, King Saud bin Abdulaziz University for Health Sciences, Jeddah, Saudi Arabia; ^7^King Abdullah International Medical Research Center, Jeddah, Saudi Arabia; ^8^College of Medicine, King Saud bin Abdulaziz University for Health Sciences, Riyadh, Saudi Arabia; ^9^Division of Pediatric Neurology, King Abdullah Specialized Children’s Hospital, Ministry of National Guard Health Affairs, Riyadh, Saudi Arabia; ^10^King Abdullah International Medical Research Center, Ministry of National Guard Health Affairs, Riyadh, Saudi Arabia; ^11^Division of Pediatric Neurology, Department of Pediatrics, College of Medicine, King Saud University, Riyadh, Saudi Arabia; ^12^King Saud Medical City, Riyadh, Saudi Arabia; ^13^PTC Therapeutics, Zug, Switzerland; ^14^King Faisal Specialist Hospital and Research Centre, Riyadh, Saudi Arabia; ^15^Neuroscience Centre, King Faisal Specialist Hospital and Research Centre, College of Medicine, Alfaisal University, Riyadh, Saudi Arabia

**Keywords:** duchenne muscular dystrophy, neuromuscular disorder, genetic diagnosis, patient demographics, Saudi Arabia, muscular dystrophy, dystrophy gene

## Abstract

Duchenne muscular dystrophy (DMD) is a rare neuromuscular disorder that is characterized by progressive muscle weakness, resulting in disability and premature death. Onset of symptoms typically occurs at 2–3 years of age, and disease progression is managed through treatment with corticosteroids. The aim of this interim analysis is to increase disease awareness and improve patient management in Saudi Arabia (SA) through the use of data from an ongoing ambispective, observational, multicenter study evaluating characteristics of patients aged 1–14 years with genetically confirmed DMD in SA. This interim analysis examined the secondary outcomes from the study–the demographics and clinical characteristics of patients included retrospectively [data recorded (enrollment visit) between January 2014 and September 2020] and prospectively between September 2020 and April 2021. The primary outcome–the list of *DMD* gene mutations for the study population–will be reported at a later date. There were 177 eligible patients. Mean, standard deviation (SD) age at enrollment was 7.5 (3.0) years. Median (min, max) age at diagnosis was 7.0 (1.3, 13.8) years. At enrollment, 28.9% of patients were full-time wheelchair users, 50.0% of ambulatory patients could run, and 63.9% could climb stairs. The mean (SD) ages of patients at enrollment who were unable to run and climb stairs were 8.0 (2.7) and 7.6 (3.0) years, respectively. Speech delay (19.4%) and learning difficulties (14.9%) were the most commonly reported intellectual impairments. Physical therapy (84.2%) was the most common choice for initial management of DMD. Only 40.7% of patients received corticosteroid therapy as part of their initial management plan, rising to 59.1% at enrollment. Devices were given to 28.8% of patients for initial management, most commonly ankle-foot orthoses (26.0%) and wheelchairs (6.2%). This analysis reports data from the largest study to date to capture demographics and clinical characteristics of DMD patients in SA. The interim results show a relatively late DMD diagnosis age compared with that in other countries, and a need for improved adherence to international DMD standard of care guidelines. Therefore, there is an urgent requirement for improved DMD education and awareness among healthcare professionals and the public in SA.

## Introduction

Duchenne muscular dystrophy (DMD) is a rare, X-linked recessive neuromuscular disorder affecting approximately 1 in every 3,500–5,000 live male births (1–3). The disease is characterized by progressive muscle degeneration, resulting in muscle weakness, motor function decline, loss of ambulation, and premature death due to respiratory or cardiac complications (1, 4, 5). DMD is caused by a mutation in the *DMD* gene, leading to absent or insufficient levels of functional dystrophin protein (1, 6, 7).

Typically, muscle weakness and walking difficulties are first noted in patients at 2–3 years of age; symptoms include toe walking, frequent falling, and difficulty with climbing stairs and running (1). Other signs and symptoms of DMD include intellectual impairment, (e.g., low IQ, learning difficulties, or autism-like behavior), lumbar lordosis and scoliosis, enlargement of the calves, and trendelenburg gait (1). Evaluation of patients suspected to have DMD includes the measurement of serum creatine kinase (CK) levels (1). Serum CK levels are elevated in patients with DMD because CK is released into the vascular system following damage to the plasma membrane of a muscle cell (8). Genetic testing is also conducted for suspected cases of DMD and is required for confirmation of a DMD diagnosis (1, 6). Physical therapy and pharmacological corticosteroid therapy, which are key aspects of the current standards of care in DMD, aim to alleviate symptoms and slow disease progression (1).

Limited information is available on how to best support the management of patients with DMD in the Middle East, including Saudi Arabia (SA). Although the international DMD care considerations published in 2018 exist to provide guidance and recommendations for the diagnosis, treatment, and management of DMD (1), an expert report summarizing the current management of DMD in the Middle East and North Africa (MENA) region concluded that clinical practice across the region was variable and disease awareness was low (9). Furthermore, limited research has been performed to investigate genetic mutations in patients with DMD in SA (10–13). Therefore, there is an unmet need for research into the demographics, clinical characteristics, and genetic mutations of patients with DMD in this region to guide future management options.

An ambispective, observational, multicenter study of patients aged 1–14 years with genetically confirmed DMD in SA is ongoing. This study is the largest of its kind performed in the Middle East and captures retrospective and prospective genetic mutation, demographics and clinical characteristics data. The aim of this interim analysis is to describe the patient demographics and characteristics data from the study to increase awareness of DMD and thereby contribute to earlier patient diagnosis and subsequent disease management in the region.

## Materials and methods

The observational study is collecting data from patients aged between 1 and 14 years with genetically confirmed DMD in routine clinical practice across nine sites in SA. Patients are included in either the retrospective or prospective arm of the study. This is an interim analysis of the study; the data cut-off point for the analysis was April 20, 2021.

### Retrospective arm

The retrospective arm of the study is complete. This included a review of the patients’ genetic testing results and medical records to identify all cases of DMD diagnosed between January 2014 and September 2020. Informed consent was not required for this arm of the study and there were no specific exclusion criteria.

### Prospective arm

The prospective arm of the study was initiated in September 2020 and is ongoing. In this arm, data are being collected from each patient with newly diagnosed, genetically confirmed DMD at one time point until the end of the study. The patients’ legal representatives must provide informed consent and patients aged 12–14 years must provide informed assent; there were no additional inclusion or exclusion criteria for the study. Clinical characteristics and patient profiles are recorded at a scheduled patient visit to their treatment center. Genetic testing results and medical records are reviewed by the site principal investigator or their research team.

### Data collection

For both the retrospective and prospective arms, data were/are collected at one time point for each patient in the study on the date when the patient visited the study site (the “enrollment visit”). All relevant data from medical records are transferred to electronic case report forms (eCRFs) by the investigator.

The primary endpoint will assess genetic mutations in the *DMD* gene, based on genetic test results performed as part of routine clinical practice for DMD diagnosis. Results for the primary endpoint will be reported when the final data analysis set is available at a later date. Secondary endpoints, which were included in this interim analysis, are as follows: the assessment of patient demographics; evaluation of clinical characteristics and patient profiles, including age at genetic diagnosis, ambulation status, ability to run and climb stairs, presence or absence of intellectual impairment (including IQ <70, speech delay, learning difficulties, and autism-like behavior), use of corticosteroids, and laboratory assessments of serum CK and alanine transaminase (ALT)/aspartate transaminase (AST) levels; and the recording of patients’ initial management plans. Place of origin data will be reported at a later date when the results for the primary endpoint are available–this will aim to identify a possible link between DMD genetic mutations and the patient’s place of origin.

The target sample size of the study, including both the retrospective and prospective arms, is approximately 150–200 patients. The sample size was based on the primary endpoint, to establish a 95% confidence interval for the prevalence of the different types of mutations on the *DMD* gene. Summary statistics will be presented for all secondary endpoints based on analysis of the eCRFs.

## Results

At the cut-off date for this interim analysis, 188 patients were enrolled; of these, 11 were excluded from the analysis owing to the following reasons: age over 14 years (*n* = 1), date of diagnosis before 2014 (*n* = 3), missing date of diagnosis (*n* = 2), and genetic test not performed [i.e., genetic data not available for these patients owing to incomplete medical records (*n* = 5)]. For the 177 patients included in the analysis, data were collected retrospectively for 170 and prospectively for 7. The mean [standard deviation (SD)] age at enrollment was 7.5 (3.0) years.

### Patient demographics and clinical characteristics

**FIGURE 1:**
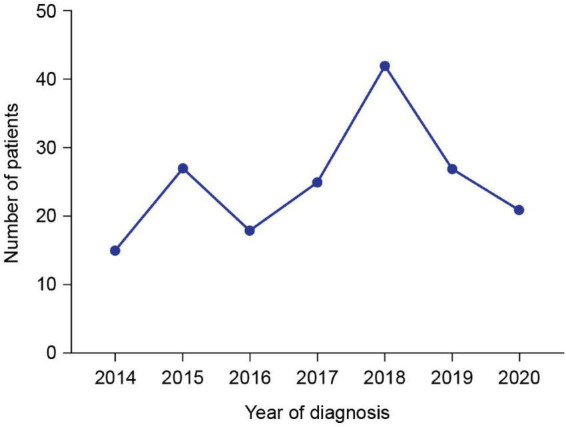
Number of patients diagnosed with DMD in each year of the study. Data for 2021 are not included because the data cut-off was on April 20, 2021.

**FIGURE 2:**
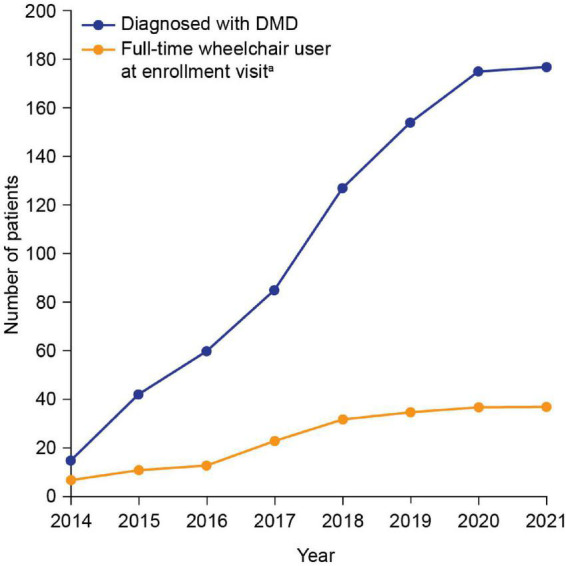
Cumulative frequency of patients diagnosed with DMD since 2014 and patients who were full-time wheelchair users at enrollment. *^a^*The enrollment visit was the date when the patient visited the study site and had data recorded. DMD, Duchenne muscular dystrophy.

Patient demographics and clinical characteristics are summarized in Table 1. Median (min, max) age at genetic diagnosis was 7.0 (1.3, 13.8) years. The number of patients diagnosed with DMD in each year of the study is shown in Figure 1. The cumulative frequency of patients diagnosed with DMD since 2014 and of patients who were full-time wheelchair users at enrollment is shown in Figure 2.

**TABLE 1:** Patient demographics and clinical characteristics.

Variable	Patients included in the interim analysis (*n* = 177)
**Age at enrollment, years**	
Mean (SD)	7.5 (3.0)
Median (min, max)	7.0 (2.0, 14.0)
**Age at genetic diagnosis, years**	
n	177
Mean (SD)	6.9 (2.7)
Median (IQR)	7.0 (5.0–8.5)
Median (min, max)	7.0 (1.3, 13.8)
**Full-time wheelchair user, *n* (%)**	
Yes	37 (28.9)
No	91 (71.1)
Data not available	49
**Patient able to run, *n* (%)**	
Yes	39 (50.0)
No	39 (50.0)
Data not available for ambulatory patients	13
Not applicable owing to being full-time wheelchair users or wheelchair-use data not available	86
**Patient able to climb stairs, *n* (%)**	
Yes	53 (63.9)
No	30 (36.1)
Data not available for ambulatory patients	8
Not applicable owing to being full-time wheelchair users or wheelchair-use data not available	86
**Intellectual impairment, *n* (%)**	
Yes	40 (29.9)
No	94 (70.1)
Data not available	43
**Corticosteroid treatment, *n* (%)**	
Yes	75 (59.1)
No	52 (40.9)
Data not available	50
**Proportion of patients with clinically abnormal laboratory assessment results, as compared with reference ranges, *n* (%)**	
ALT	115/119 (96.6)
AST	103/103 (100)
CK	146/146 (100)

ALT, alanine transaminase; AST, aspartate transaminase; CK, creatine kinase; DMD, duchenne muscular dystrophy; IQR, interquartile range; SD, standard deviation.

**FIGURE 3:**
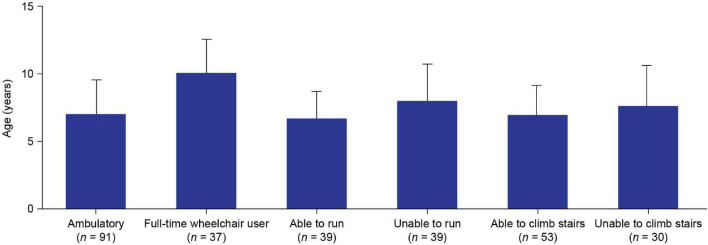
Mean (SD) age of: ambulatory and full-time wheelchair user patients at enrollment^a^; and ambulatory patients who were able and unable to run or climb stairs at enrollment. ‘Ambulatory’ or “wheelchair-use” data were not available for 49/177 patients. “Ability to run” data were not available for 13/91 ambulatory patients. “Ability to climb stairs” data were not available for 8/91 ambulatory patients. *^a^*The enrollment visit was the date when the patient visited the study site and had data recorded. SD, standard deviation.

Of the 128 patients with available data, 91 (71.1%) were ambulatory and 37 (28.9%) were full-time wheelchair users at the enrollment visit; mean (SD) patient age was 7.0 (2.5) and 10.1 (2.5) years for ambulatory patients and full-time wheelchair users, respectively (Figure 3). Of the ambulatory patients with available data, 39/78 (50%) were able to run and 53/83 (63.9%) were able to climb stairs at the enrollment visit. The mean age of patients who were able to run and climb stairs was 6.6 (2.0) and 6.9 (2.2) years, respectively, and the mean age of those who were unable to run and climb stairs was 8.0 (2.7) and 7.6 (3.0) years, respectively. Speech delay (19.4%) and learning difficulties (14.9%) were the most commonly reported intellectual impairments. Treatment with corticosteroids was reported in 59.1% of patients. The proportions of patients with clinically abnormal laboratory assessment results for ALT, AST, and CK levels, as compared with reference ranges of the 9 sites, were 96.6% (115/119), 100% (103/103), and 100% (146/146), respectively (Table 1).

### Initial management plans

**FIGURE 4:**
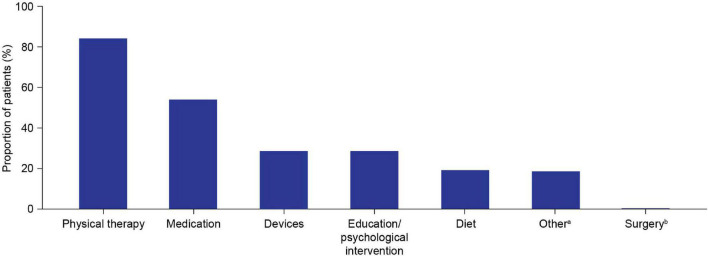
Initial patient management plan components. Multiple components could be selected for each patient. ^a^Other initial management plan components included the following categories, which group together free-text responses for initial management plans that were not covered by the six predefined options: cardiac evaluation/follow-up/management (*n* = 26); bone health management (*n* = 19); genetic counseling/carrier testing (*n* = 3); occupational therapy (*n* = 2); speech therapy (*n* = 2); neurological referral/management (*n* = 3); orthopedic referral (*n* = 1); referral to general practitioner (*n* = 1). ^b^Spine surgery/thoracic lumbar fusion.

Physical therapy (84.2%) was the most commonly reported choice for initial management, followed by medication (54.2%) (Table 2 and Figure 4). The most frequently reported initial medications were corticosteroids (40.7%) and vitamin D (39.0%) (Table 2). The most commonly used devices for initial management were ankle-foot orthoses (26.0%) and wheelchairs (6.2%) (Table 2).

**TABLE 2:** Initial patient management plans^a^.

Initial management plan, *n* (%)	Patients included in the interim analysis (*n* = 177)
Medication	96 (54.2)
Physical therapy	149 (84.2)
Devices	51 (28.8)
Diet	34 (19.2)
Educational and psychological interventions	51 (28.8)
Surgery (spine surgery/thoracic lumbar fusion)	1 (0.6)
Other^a,b^	33 (18.6)
**Medication**	
Vitamin D supplements	69 (39.0)
Omeprazole	7 (4.0)
Ataluren	9 (5.1)
Calcium carbonate	32 (18.1)
Corticosteroids	72 (40.7)
**Devices**	
Ankle-foot orthoses	46 (26.0)
Wheelchair	11 (6.2)
Medical shoe	5 (2.8)

^a^Multiple components could be selected for each patient.

^b^Other initial management plan components included the following categories, which group together free-text responses for initial management plans that were not covered by the six predefined options: cardiac evaluation/follow-up/management (*n* = 26); bone health management (*n* = 19); genetic counseling/carrier testing (*n* = 3); occupational therapy (*n* = 2); speech therapy (*n* = 2); neurological referral/management (*n* = 3); orthopedic referral (*n* = 1); referral to general practitioner (*n* = 1).

## Discussion

This interim analysis of the largest study of DMD patients in SA to date provides initial insights into demographics and clinical characteristics data from 177 patients diagnosed between 2014 and 2021. A slight increase in the number of DMD diagnoses over time was observed–this is encouraging and may demonstrate an increase in DMD awareness in the region. There was a peak in the number of diagnoses in 2018, perhaps due (at least in part) to an increase in disease awareness after the publication of the DMD care considerations, which were available online from January 2018 (1); however, the number of diagnoses decreased in the following years. The drop in the number of diagnoses was likely owing to the closure of most of the hospital outpatient clinics during the COVID-19 pandemic.

The mean age at genetic diagnosis in SA was 6.9 years, which is later than the typical onset of signs and symptoms of DMD at approximately 2–3 years of age (1). This observation is concordant with a previously published expert opinion on the situation in the MENA region, which reported a mean age at genetic diagnosis of 7–8 years (9). A mean age at diagnosis of 6.9 is 2.6 years later than that reported by the CARE-NMD project (which evaluated the care practices for 1,062 patients with DMD in seven European countries between 2011 and 2012 using a cross-sectional survey of patients) (14) and 1.7 years later than that reported as of 2018 by the Strategic Targeting of Registries and International Database of Excellence (STRIDE) Registry [an ongoing, multicenter, observational, post-approval safety study of the mutation-specific therapy ataluren in patients with nonsense mutation DMD (nmDMD)] (15).

Therefore, results from the present analysis indicate that the diagnosis of DMD in SA is relatively late compared with that in other countries. Although some patients with DMD in SA are diagnosed early if they are referred to a specialist early, these results suggest that further education is needed in the region to prompt primary care physicians to more urgently refer patients with suspected DMD to specialists. Late diagnosis indicates a missed opportunity to provide appropriate treatment early in the course of the disease, which is important for slowing disease progression, improving patient outcomes and increasing life expectancy (1). Delays in diagnosis could be due to a lack of awareness of DMD leading to a delay in genetic testing/screening for CK levels, or a lack of resources for diagnosing DMD (e.g., limited access to genetic testing) (9). Given that genetic testing has become more widely available in SA over the past 3–5 years, it is likely that the primary reason for delays in diagnosis is the lack of clinical recognition by primary care providers, especially at the early stages of the disease course.

The mean age at enrollment of ambulatory patients and patients who were full-time wheelchair users in this study was 7.0 and 10.1 years, respectively. This indicates a period of significant DMD disease progression between these ages. This finding is further supported by the mean ages of patients who were able to run or climb stairs (6.6 and 6.9 years, respectively) being approximately 1 year lower than the mean ages of those who were unable to run or climb stairs (8.0 and 7.6 years, respectively). These data support previous observations of a phase of rapid decline in motor function over the period of approximately 1 year, which starts sometime after the age of 7 years (9).

It is recommended that physical therapy should begin as early as possible following DMD diagnosis (1); the most common initial management plan reported here was physical therapy (84.2%). Although this is encouraging, there is more to be done to ensure that all patients receive physical therapy as part of their initial management plan. Corticosteroids are recommended as a standard of care (SoC) treatment for use in patients with DMD from the early ambulatory stage of disease progression, before significant physical decline (1, 9).

Just over 40% of patients were prescribed corticosteroids as part of their initial treatment management plan. However, at study enrollment the proportion of patients treated with corticosteroids had risen to almost 60%. While it is reassuring that the uptake of corticosteroids increased between diagnosis and study enrollment, it is concerning that the proportion is not substantially higher in a population that is declining rapidly in motor function. The proportion of DMD patients in SA treated with corticosteroids is also considerably lower than in other regions; for example, the Cooperative International Neuromuscular Research Group Duchenne Natural History Study, which took place at 20 study centers across nine countries between 2006 and 2016, reported that 87% of patients received corticosteroid treatment (16), and the STRIDE Registry reported that 89.2% of patients received corticosteroid treatment (15). The reasons for low corticosteroid use in SA are not clear, but may include region-specific concerns in the Middle East regarding corticosteroid side effects (9, 17).

This interim analysis has some limitations. As the first analysis of the study, the interim analysis captured patients’ clinical characteristics and motor function data at the enrollment visit only and did not include a longitudinal follow-up of the patients. Therefore, it is not yet possible to monitor changes in disease progression over time. Given that ages at loss of ambulation and ages at decline of other motor function were not recorded, it will not be possible to determine mean ages at decline in motor function. Moreover, as this manuscript reports interim data as of April 20, 2021, it includes some missing data (e.g., 49/177 patients had missing data for ambulation status); these missing data however will be included in the final report. Another limitation is that the initial management plans documented in the analysis may not be comprehensive because the study investigators were only able to record certain management plans (e.g., cardiac management) by entering free-text responses in the “other” category.

Overall, the findings highlight the following unmet needs: (1) improvement of disease awareness and diagnostic tools to support earlier diagnosis; (2) provision of guidance and education to healthcare providers, patients and caregivers about treatment and management options; and (3) further research into the barriers to prescribing recommended therapies, such as corticosteroids. One practical recommendation is to create national guidelines designed for SA to be used by pediatricians, who are the main point of contact for DMD patients given the lack of pediatric neurologists across the country.

In conclusion, these interim data from the largest study of patients with DMD in SA to date provide insights into the demographics and clinical profiles of patients and highlight unmet needs regarding the diagnosis, treatment, and management of patients with DMD. Increasing awareness and education on these topics could be a first step toward improving patient outcomes.

## Data availability statement

The original contributions presented in this study are included in the article/supplementary material, further inquiries can be directed to the corresponding author.

## Ethics statement

Local IRB approval was granted for patients in the prospective arm. Ethical review and approval was not required for the participants in the retrospective arm in accordance with the local legislation and institutional requirements. Informed consent to participate in this study was provided by the participants’ legal guardian/next of kin.

## Author contributions

AzA, FA, AB, NA, MMq, OM, FB, BA, AhA, and MMh were investigators for the study and were involved in the study design and data collection. AM and EH were involved in the study design and data collection. All authors contributed to the writing of the article, reviewed all drafts, and approved the submitted version. The concept for the manuscript was agreed between the authors and PTC Therapeutics, and the decision to submit the manuscript for publication was made by the authors and PTC Therapeutics.

## Abbreviations

ALT: alanine transaminase
AST: aspartate transaminase
eCRF: electronic case report form
CK: creatine kinase
DMD: Duchenne muscular dystrophy
MENA: Middle East and North Africa
nmDMD: nonsense mutation DMD
SA: Saudi Arabia.
